# Metabolites and Their Bioactivities from the Genus *Cordyceps*

**DOI:** 10.3390/microorganisms10081489

**Published:** 2022-07-24

**Authors:** Shuai-Ling Qu, Su-Su Li, Dong Li, Pei-Ji Zhao

**Affiliations:** State Key Laboratory for Conservation and Utilization of Bio-Resources in Yunnan, School of Life Sciences, Yunnan University, Kunming 650091, China; slqu1998@outlook.com (S.-L.Q.); ssl1005@outlook.com (S.-S.L.); ddll1998@outlook.com (D.L.)

**Keywords:** *Cordyceps*, *Cordyceps sinensis*, metabolites, bioactivity

## Abstract

The *Cordyceps* genus is a group of ascomycete parasitic fungi, and all known species of this genus are endoparasites; they mainly feed on insects or arthropods and a few feed on other fungi. Fungi of this genus have evolved highly specific and complex mechanisms to escape their host’s immune system and coordinate their life cycle coefficients with those of their hosts for survival and reproduction; this mechanism has led to the production of distinctive metabolites in response to the host’s defenses. Herein, we review approximately 131 metabolites discovered in the genus *Cordyceps* (including mycelium, fruiting bodies and fungal complexes) in the past 15 years, which can be used as an important source for new drug research and development. We summarize chemical structures, bioactivity and the potential application of these natural metabolites. We have excluded some reports that originally belonged to *Cordyceps*, but whose taxonomic attribution is no longer the *Cordyceps* genus. This can and will serve as a resource for drug discovery.

## 1. Introduction

*Cordyceps sinensis* is a renowned Chinese herbal medicine and has been widely used for medicinal treatment in China for over 300 years [1]. *C. sinensis* grows in very limited habitats, its yields decrease year by year, and its use is finite because of its high price and limited availability [2]. Researchers have been seeking substitute materials by investigating the fermentation and culture of fungi separated from *C. sinensis* and other *Cordyceps* species [3]. *Cordyceps* is the most numerous and diverse genus of the Clavellaceae family, of which 629 species have been identified, according to MycoBank (https://www.mycobank.org; accessed on 8 June 2022). It is a class of ascomycete parasitic fungi. All known species act as endoparasites, feeding mostly on insects and other arthropods and a little on other fungi. This survival mechanism of *Cordyceps* leads to the production of distinctive metabolites in response to host defenses, which is an important source for new drug research and development [4,5]. There are many species of *Cordyceps*. They are abundant in humid climates and tropical forests, widely distributed in North America; Europe; East and Southeast Asian countries, especially China, Japan, Nepal, Vietnam, Bhutan, Korea and Thailand, although some other species are also found in different habitats in other regions, indicating a global distribution. Species in the genus *Cordyceps* are widely accepted for using as food and medicine and good reviews have been published. For example, a review published by Zhou et al. reported on natural products, pharmacological functions, and novel products in *Cordyceps sinensis* [6]. Similarly, Olatunji et al. reviewed the advanced developments in traditional uses, phytochemistry and pharmacology of *Cordyceps* fungi, with a primary focus on *C. sinensis* and *C. militaris* [7]. On the other hand, Chen et al. provided an overview on the safety concerns of fungal fruiting bodies of several *Cordyceps* species in terms of their existence as food supplements or as animal feed by-products and analyzed the conservatism of gene clusters between *Cordyceps* and other fungi involved in toxin production [8]. In the past 15 years, researchers have reported many new strains and active metabolites, and the development of biotechnology has provided more possibilities for investigating these novel and diverse metabolites. Moreover, many polysaccharides have been isolated and identified in *Cordyceps* and have showed various biological activities. Due to the taxonomic characteristics of fungi, some metabolites of fungi that previously belonged to the genus *Cordyceps* but are no longer characterized as *Cordyceps* are often cited. We will summarize the bioactive compounds from the *Cordyceps* genus that have been reported in the literature since 2007. The species of the genus *Cordyceps* present an important source of therapeutic effect for various diseases due to the presence of bioactive components, which can serve as potential leads for drug discovery.

## 2. Nucleosides and Their Activities

**Figure 1:**
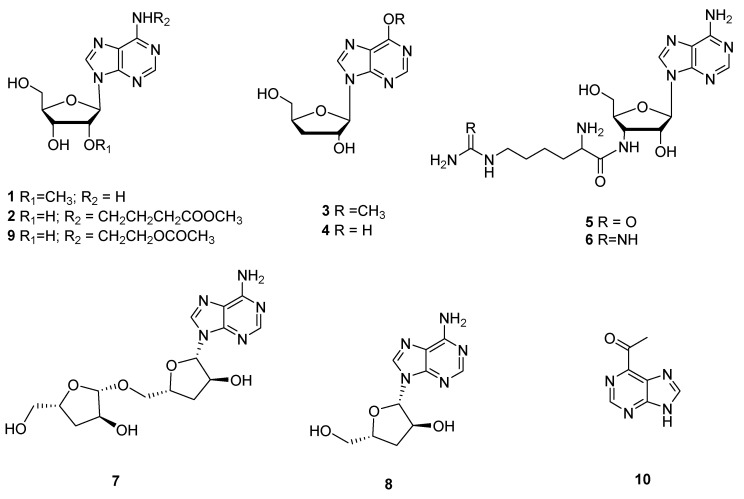
Nucleosides (**1**–**10**) from *Cordyceps*.

Nucleoside compounds are the main metabolites and active components of *Cordyceps* fungi. Cordysinin B (**1**) was separated from the mycelium of *C. sinensis* and inhibited the formation of superoxide anions and the release of elastase with an IC_50_ value of 0.15 μM [9]. Two new nucleosides, N6-4-methylbutyrat-adenosine (**2**) and 3′-deoxy-6-*O*-methylinosine (**3**), were isolated from *C. militaris* and did not show anti-inflammatory inhibitory activity under 100 μM [10]. 3’-Deoxyinosine (**4**) was isolated from *C. militaris* and showed a weak toxic effect on cancer cells A549, PANC-1 and McF-7 and exhibited powerful cytotoxicity against MCF-7 cells at a concentration of 30 μM [11]; moreover, it was previously reported to have antigenic activity. In addition, when compound **4** was combined with doxorubicin, it inhibited the metastasis and proliferation of breast cancer cells. According to the Chou-Talalay method, when 80 μmol/L 3’-deoxyinosine (**4**) and 1 μmol/L doxorubicin were used together, the synergistic effect was the strongest, and the CI value and the cell inhibition rates were 0.665 and 60.31 ± 1.06%, respectively [12]. Two metabolites, (2-amino-N-((2*S*,3*S*,4*R*,5*R*)-5-(6-amino-9*H*-purin-9-yl)-4-hydroxy-2-(hydroxymethyl)–tetrahydrofuran-3-yl)-6-ureido-hexanamide) (**5**) and (2-amino-N-((2*S*,3*S*,4*R*,5*R*)-5-(6-amino-9*H*-purin-9-yl)-4-hydroxy-2-(hydroxymethyl)tetrahydrofuran-3-yl)-6-guanidinohexanamide) (**6**), were identified from *C. militaris*. Anti-tumor experiments showed that compounds **5** and **6** considerably inhibited the propagation of HepG2 liver cancer cells at 72 h and their IC_50_ values were 0.09 μM and 0.51 μM, respectively [13]. A new ribonucleotide of 5’-(3’’-deoxy-β-D-ribofuranosyl)-3’-deoxyadenosine (**7**) as well as a known 3’-deoxyadenosine (**8**) were isolated from the *C. militaris*; 3’-deoxyadenosine **8** was shown to inhibit the expression of the NF-κB reporter gene in HeLa cells induced by TNF at concentrations of 3–100 μM, but compound 7 showed no inhibitory activity [14]. A new nucleoside cordyrrole B (**9**), an acetylated form of N6-(2-hydroxyethyl)adenosine, was obtained from the active components of *C. militaris* and showed the significant inhibition of pancreatic lipase activity at 100 μM [15]. 6-Acetylpurine (**10**) was isolated from *C. militaris* [16]. The structures of **1**–**10** are shown in Figure 1.

Cordycepin is a vital active component of *Cordyceps*. During in-depth research on cordycepin, it was found that cordycepin has a wider range of biological activities, and many new mechanisms of action were discovered. Due to the emergence of mutant strains, the coronavirus disease 2019 (COVID-19) is ongoing globally, often causing severe acute respiratory syndrome and leading to the death of some patients. Using computational methods, researchers predicted a possible inhibitory affinity of cordycepin to the main SARS-CoV-2 protein target [17]. The latest research shows that cordycepin can effectively inhibit the reproduction of new SARS-CoV-2 drug-resistant strains, and its EC_50_ was about 2 μM in in vitro anti-SARS-CoV-2 assays, which is superior to remdesivir and its active metabolite GS-441524 [18]. In addition, it was found that cordycepin can inhibit Dengue virus replication and significantly reduced DENV protein at EC_50_ of 26.94 μM [19]. Other research on the activity of cordycepin found it could protect PC12 cells from the neurotoxicity induced by 6-hydroxydopamine through its powerful antioxidant activity [20]. Additionally, cordycepin modulated adenosine A1 receptors to increase long-term enhancement-capability formation and neuronal survival in the BCCAO model and glutamate-enticed HT22 neuronal cell death via the p38/JNK/ERK pathway [21,22]. In recent years, progress has been made in the study of the anti-cancer mechanisms of cordycepin, and many reviews show that cordycepin may facilitate tumor cell death via cysteine–aspartic proteases (caspases), mitogen-activated protein kinase (MAPK) and glycogen synthase kinase (GSK)-3β pathways mediated by putative adenosine receptors, death receptors and/or epidermal growth factor receptors (EGFR) [23,24]. In particular, cordycepin regulates the phosphoinositide 3-kinase/protein kinase B (PI3K/AKT) signaling pathway and inhibits cyclin-dependent kinase 2 (Cdk-2), extracellular signal-regulated kinase 1/2 (ERK1/2) and Rb/E2F1 and fibroblast growth factor receptors 1–4 (FGFR 1–4) regulate the cell cycle and further reduce the growth of testicular tumors, gastric cancer cells and cervical cancer cells [25,26]. In addition, cordycepin also regulates diverse signaling proteins, such as hedgehog, glioblastoma protein (GLI), DNA-dependent protein kinase (DNA-PK) and ERK, to induce cancer cell apoptosis [27,28].

## 3. Non-Ribosomal Peptides and Alkaloids

Three new cordysinins C-E (**11**–**13**) were isolated from the mycelia of *C. sinensis*, but showed no antioxidant activity even at 500 μM [9]. Two new pyrrole alkaloid derivatives, 2-carboxaldehyde-1-(4-aminobutyl-5-(methoxymethyl)-1*H*-Pyrrole (**14**), and 2-carboxaldehyde-5-(methoxymethyl)-1-(2-oxo-3-piperidinyl)-1*H*-pyrrole (**15**), were separated from *C. militaris*, and the anti-inflammatory effects results showed no significant inhibitory activity higher than 100 μM [10]. A new alkaloid cordyrrole A (**16**) was separated from the extract of *C. militaris* and demonstrated the inhibition of adipocyte differentiation and pancreatic lipase activity at a concentration of 100 μM [15]. Two new alkaloid metabolites, cordytakaoamides A (**17**) and B (**18**), were isolated from cultures of *C. takaomontana* NBRC 101754, and the absolute configuration of cordytakaoamides A and B was expounded by the total synthesis of cordytakaoamide A and the experimental circular dichroism [29]. Five new alkaloids, cordycepamides A-E (**19**–**23**) were isolated from *C. sinensis*. Cordycepamide D (**22**) showed moderate radical scavenging activity, its IC_50_ value concentration was 51.42 ± 3.08 μM in an analysis of DPPH [30]. Three new macrocyclotetralactamides, gunnilactams A-C (**24**–**26**), were separated from the deep fermentation liquid of *C. gunnii*; **25** and **26** were isomers. Gunnilactam A (**24**) was selectively cytotoxic against human prostate cancer C42B cells and its IC_50_ was 5.4 μM [31].

**Figure 2:**
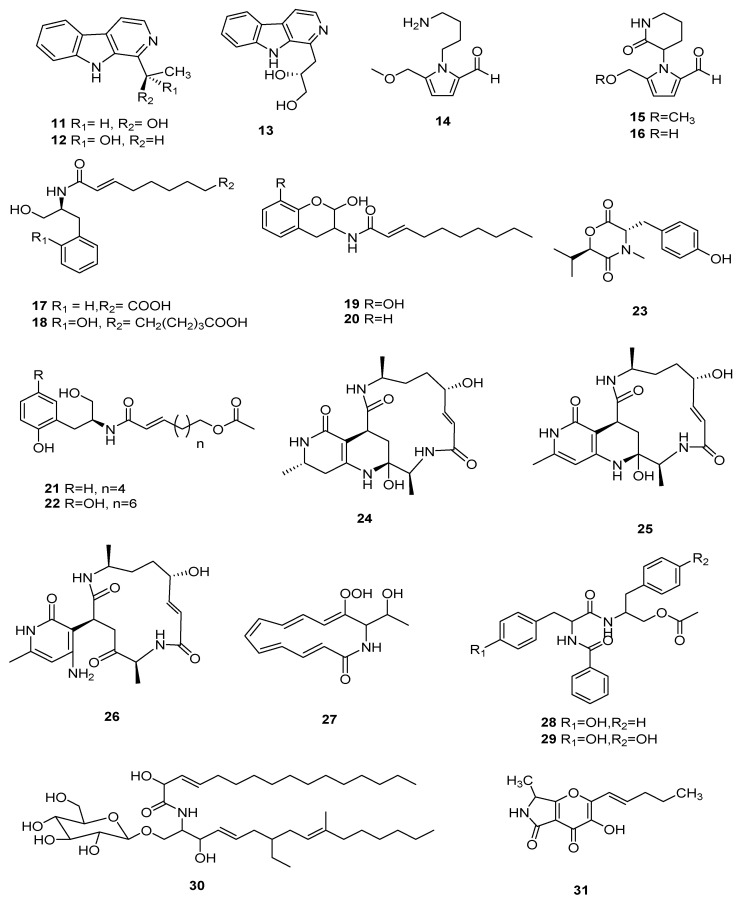
Non-ribosomal peptides and alkaloids (**11**–**31**) from *Cordyceps*.

A cordycepic pigment cordycepene (**27**) was identified from *C. militaris*, and it had remarkable DPPH radical-scavenging activity; the IC_50_ was 0.81 mg/mL in antioxidant assays. In anti-aging assays, the activities of CAT, GSH-Px and SOD increased by 201.05%, 708.26% and 341.50%, respectively, and the degree of MDA decreased by 29.92%, when the concentration of compound **27** enhanced from 0 to 50 μg/mL [32]. Two new compounds, cordyceamides A (**28**) and B (**29**), were separated from *C. sinensis* (BERK.) SACC. Compounds **28** and **29** had cytotoxic effects on the cells of lines A375, L929 and Hela [33]. A new cerebroside, cordycerebroside B (**30**), was isolated from *C. militaris* and showed remarkable inhibition activity against PTP1B with an IC_50_ value of 4.68 ± 0.18 μM [34]. A new lactam-fused 4-pyrone cordylactam (**31**) was obtained from the *Cordyceps* sp. BCC 12671; however, its biological activity was not tested [35]. The structures of **11**–**31** are shown in Figure 2.

**Figure 3:**
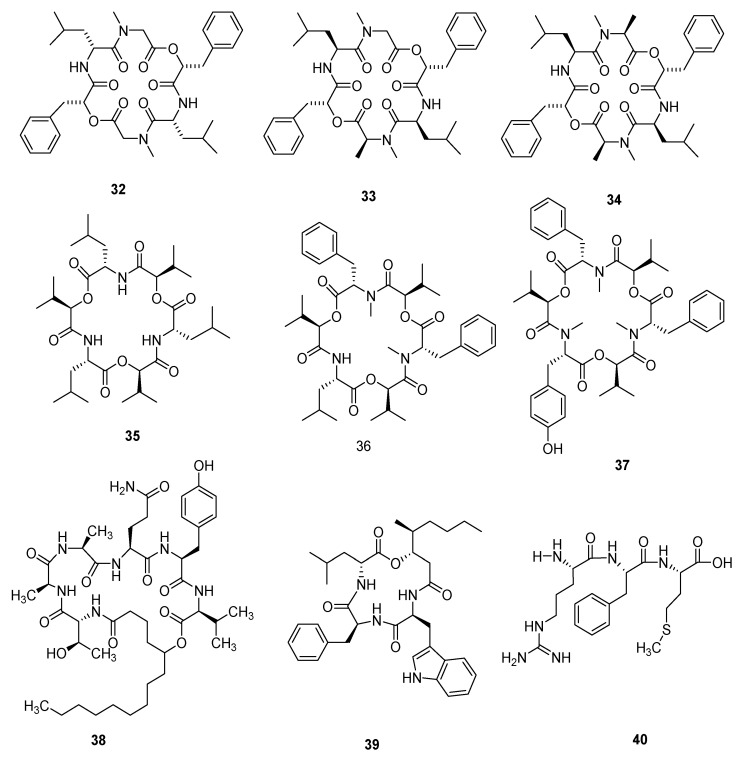
Non-ribosomal peptides (**32**–**40**) from *Cordyceps*.

Cardinalisamides A–C (**32**–**34**) were separated from *C. cardinalis* NBRC 103832. Cardinalisamides A–C showed antitrypanosomal activity against *Trypanosoma brucei* and their IC_50_ were 8.56, 8.65 and 8.63 mg/mL, respectively, in vitro, and had IC_50_ values of 18.48, 14.00 and 23.84 mg/mL, respectively, against normal human diploid fibroblasts (MRC-5 cells) in cytotoxicity assays [36]. Cordycecin A (**35**) and two known compounds beauvericins E (**36**) and J (**37**) were separated from the ascocarps and insect-body tranches of *C. cicadae*. Beauvericin J (**37**) was cytotoxic against HepG2 and HepG2/ADM cells, whose IC_50_ values were in the range of 5.04 ± 0.20 μM and 2.67 ± 0.09 μM, respectively; beauvericin E (**36**) revealed moderate inhibitory effects and its IC_50_ value was in the range of 13.67 ± 2.59 μM and 14.48 ± 1.68 μM, respectively [37]. Cordycommunin (**38**) was separated from the *Ophiocordyceps communis* BCC 16475 and it had a growth inhibiting effect on *Mycobacterium tuberculosis* H37Ra and its MIC value was 15 µM. Compound **38** also exhibited slight cytotoxicity to KB cells and its IC_50_ value was 45 µM [38]. Beauverolide J_b_ (**39**) was obtained from *C. javanica* [39]. A tripeptide, arginylphenylalanyl-methionine (**40**), isolated from *C. gunnii*, with the molecular formula of C_20_H_32_N_6_O_4_S, is a sedative and hypnotic active substance [40]. Other polypeptide compounds have had less report published in recent years. However, recent studies have shown that cordyceps polypeptide complexes are helpful for improving learning and memory, but their mechanism of action needs further study [41]. The structures of **32**–**40** are shown in Figure 3.

## 4. Polyketides

Three new compounds, paecilomycones A-C (**41**–**43**), were identified from a methanol extract of *C. gunnii*. Paecilomycones A-C showed significant tyrosinase inhibitory activity, and the IC_50_ values were 0.11 μM, 0.17 μM and 0.14 μM, respectively. Structure and activity research showed that the tyrosinase inhibition activity was connected to the amount of hydroxyl groups on the paecilomycones.

The structure of compound **41** is very similar to the anti-HIV target flurane (anti-HIV, the IC_50_ value was 1.7 μM), while the compound **43** has a NH_2_ group in C-9 rather than the usual -OH group, which indicates that compounds **41** and **43** may be promising anti-HIV products with high importance for follow-up studies [42]. Two new compounds, 2-(3-carboxy-2-hydroxypropyl)-3-methyl-2-cyclopentenone (**44**) and 5-(2- hydroxyethyl)-2-furanacetic acid (**45**), were isolated from the *C. cicadae* mycelia [43]. Two compounds, rugulosin (**46**) and skyrin (**47**), were identified from *C. formosana*; the LD_50_ of compounds **46** and **47** were 18.3 ± 0.2 and 103.7 ± 5.9 μg/mL against CHO cells in cytotoxic assays [44]. Opaliferin (**48**), which has a novel C19 skeleton, was separated from the *Cordyceps* sp. NBRC 106954. Opaliferin showed slight cytotoxicity against HeLa, HSC-2 and RERF-LC-KJ tumor cell lines at 100 μM, and the inhibitory rates were 60%, 30% and 20%, respectively [45].

**Figure 4:**
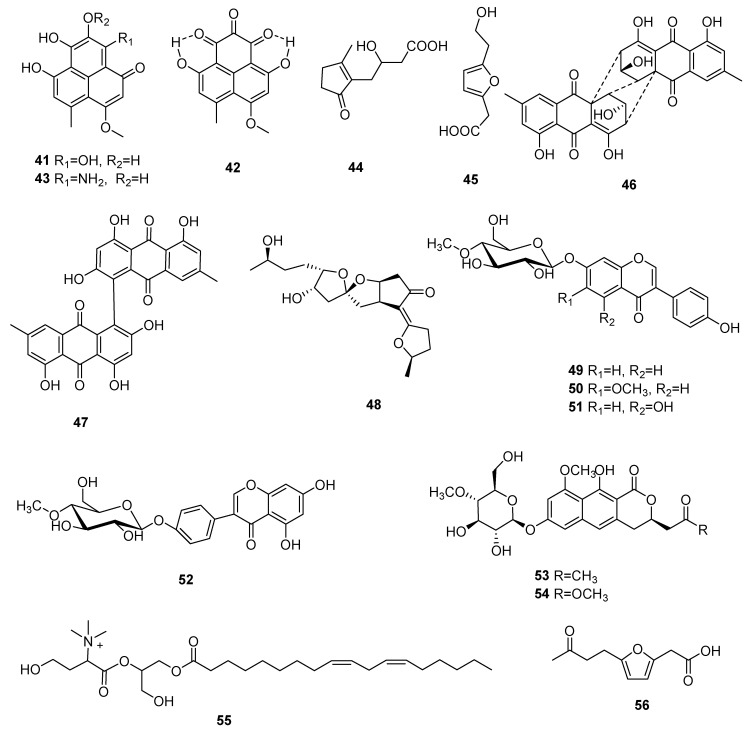
Polyketides (**41**–**56**) from *Cordyceps*.

Four novel isoflavone methyl-glycosides, daidzein 7-O-β-D-glucoside 4″-*O*-methylate (**49**), glycitein 7-*O*-β-D-glucoside 4″-*O*-methylate (**50**), genistein 7-*O*-β-D-glucoside 4″-*O*-methylate (**51**) and genistein 4′-*O*-β-D-glucoside 4″-O-methylate (**52**) were obtained from *C. militaris* which was grown on germinated soybeans. Compound **52** showed marked antiallergic activity [46]. Three new compounds fumosoroseanosides A (**53**) and B (**54**) and fumosoroseain A (**55**) were isolated from *C. fumosorosea*. At experimental doses of 10–30 μg, compounds **53** and **54** exhibited excellent (*p* < 0.05) antibacterial and antifungal activity compared to the negative control group. In a range of concentrations from 100 μM to 400 μM, compound **55** could lengthen the life of nematodes; at 200 μM, it showed optimal activity (*p* < 0.05), and the average life of the nematodes was lengthened by 11.3% [47]. The structures of **41**–**56** are shown in Figure 4.

**Figure 5:**
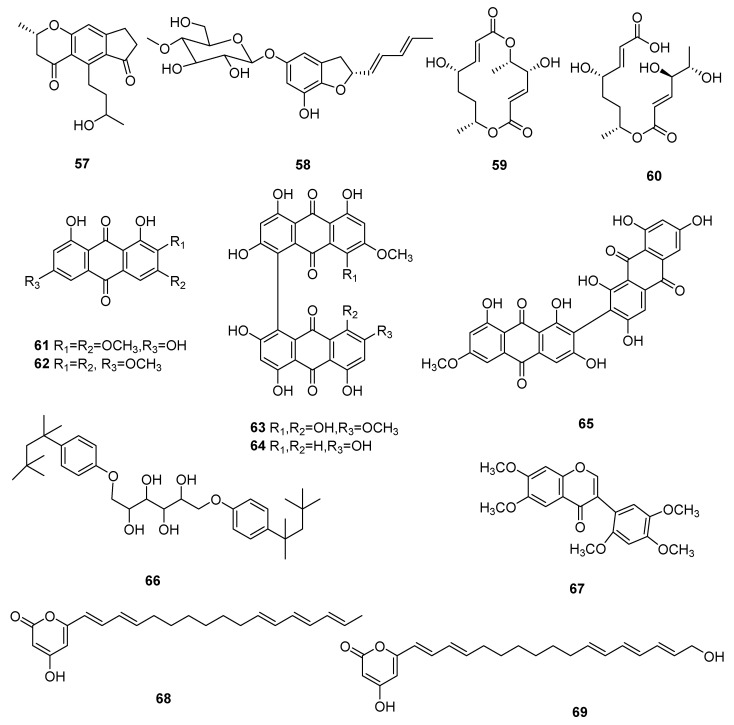
Polyketides (**57**–**69**) from *Cordyceps*.

Two new compounds 2-(5-(3-oxobutyl) furan-2-yl) acetic acid (**56**) and cordycepone (**57**) were isolated from *Cordyceps* spp., a strain formed by cell fusion in *Cordyceps militaris* and *Cordyceps cicadae*. Compound **56** showed a slightly inhibiting effect on AChE, and its inhibition rate was 16.41% [48]. A new glycosylated asperfuran (**58**) was isolated from *C. javanica*, whose biological activity has not been reported [39]. A new stereoisomer of clonostachydiol, cordybislactone (**59**), and its hydrolyzed derivative (**60**), were obtained from *Cordyceps* sp. BCC 49294 [49], whose biological activity has not been reported. Five new anthraquinones, morakotins A–E (**61**–**65**), were purified from the *C. morakotii* BCC 56811 and demonstrated antimicrobial activity. Morakotin C (**63**) showed moderate antifungal activity against *Candida albicans* (the value of IC_50_ was 25.87 µg/mL) and antibacterial effect on *Bacillus cereus* (the value of MIC was 12.5 µg/mL). Morakotin D (**64**) showed marked antibacterial activity against *B. cereus* (the value of MIC was 3.13 µg/mL) and *Staphylococcus aureus* (the value of MIC was 6.25 µg/mL) [50]. A novel derivative MA-1 [(2*R*,3*R*,4*R*,5*R*)-1,6-bis(4-(2,4,4-trimethylpentan-2-yl)phenoxy)hexane-2,3,4,5-tetraol] (**66**), was synthesized from the constituents of *C. militaris*. MA-1 (**66**) demonstrated powerful anti-cancer potential for the cure of H358, A549 and H460 cell lines, and its value of IC_50_ was 5 μM for A549 human lung cancer cells [51]. 6,7,2′,4′,5′-Pentamethoxyflavone (**67**) was isolated from *C. militaris* and its inhibition of hemolysis was 92.08 ± 1.85% at 250 μg/mL [52]. Cordypyrones A (**68**) and B (**69**) were isolated by the heterologous expression of a biosynthetic gene cluster from *C. militaris* in *Aspergillus nidulans*. Cordypyrones A (**68**) and B (**69**) showed moderate inhibitory effects on ATCC 9372 and both of their MIC values were 16 µg/mL; the MIC value were 16 and 8 µg/mL, respectively, against ATCC 49064; and the MIC values were 32 µg/mL against ATCC 25922 [53]. The structures of **57**–**69** are shown in Figure 5.

## 5. Sterols and Terpenoids

It has been reported that sterols in *C. sinensis* have anti-tumor activity, immunosuppression, anti-arteriosclerosis and antibacterial activities. However, the active sterols in *C. militaris* are seldom reported.

**Figure 6:**
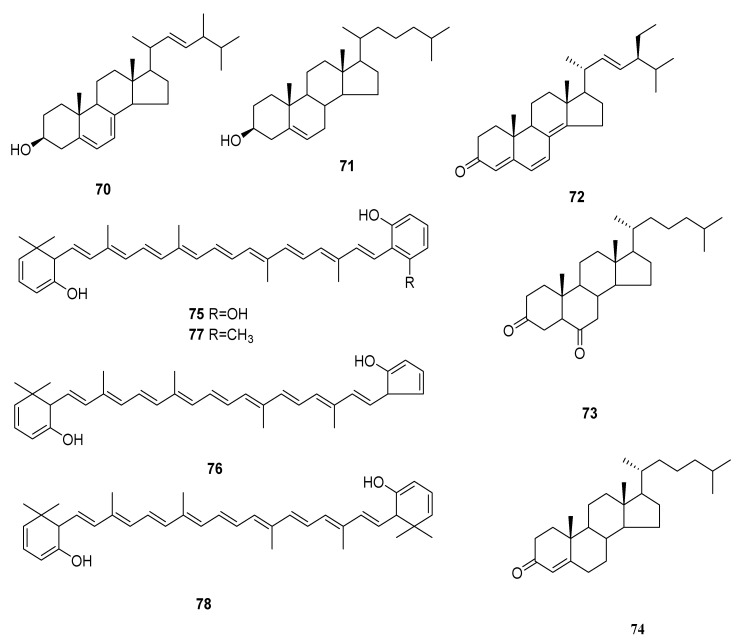
Sterols and terpenoids (**70**–**78**) from *Cordyceps*.

Reports from 2015 showed a new anti-cancer compound named (3*R*,9*R*,10*S*,13*S*,14*S*,17*S*)-17-((2*S*,5*R*,*E*)-5,6-dimethylhept-3-en-2-yl)-10,13-dimethyl-2,3,4,9,10,11,12,13,14,15,16,17-dodecahydro-1*H*-cyclopenta[α]phenanthren-3-ol (**70**), was purified from *C. militaris*. Within 48 h, compound 70 exhibited dosage-dependent and time-dependent inhibition on the growth of A549 cells, and lung cancer cells were almost no longer growing at a concentration of 120 μg/mL. These results revealed that compound 70 was likely to inhibit the proliferation of human lung cancer A549 cells [54]. Cholest-5-en-3β-ol (**71**), 22-tetraen-3-one (**72**), 5α-cholest-3,6-dione (**73**) and cholest-4-en-3-one (**74**) were separated from *C. militaris* for the first time [14]. Four novel carotenoids were separated from *C. militaris* and confirmed as xanthophylls and their names were cordyxanthin-I (2,3,2′,3′-tetradehydro-18,16′,17′,18′-tetranor-ε,ε-carotene-5,5′,1′-triol) (**75**), cordyxanthin-II (2,3,2′,3′-tetradehydro-18,1′,16′,17′,18′-pentanor-ε,ε-carotene-5,5′,-diol) (**76**), cordyxanthin-III (2,3,2′,3′-tetrade hydro-18,17′,18′-trinor-ε,ε-carotene-5,5′,-diol) (**77**) and cordyxanthin-IV (2,3′,3′-tetradehydro-18,18′-dinor-ε,ε-carotene-5,5,-diol) (**78**) [55]. The structures of **70**–**78** are shown in Figure 6.

## 6. Aromatics and Their Derivatives

Five new aromatics bearing a 4-*O*-methylglucose unit, named 3-methoxy- 1,4-hydroquinone 1-(4′-*O*-methyl-β-glucopyranoside) (= 4-hydroxy-3-methoxy phenyl 4-*O*-methyl-β-glucopyranoside) (**79**), 3-methoxy-1,4-hydroquinone 4-(4′-*O*-methyl-β-glucopyranoside) (=4-hydroxy-2-methoxyphenyl 4-*O*-methyl-β-glucopyranoside) (**80**), vanillic acid 4-(4′-*O*-methyl-β-glucopyranoside) (=3-methoxy-4-[(*O*-methyl-β-glucopyranosyl) oxy]benzoic acid) (**81**), 5-methoxycinnamic acid 3-*O*-(4′-*O*-methyl-β-glucopyranoside) (=(2*E*)-3-[3-methoxy-5-[(4-*O*-methyl-β-glucopyranosyl)oxy]phenyl]prop-2-enoic acid (**82**) and naphthalene-1,8-diol 1,8-bis(4′-*O*-methyl-β-glucopyranoside) (=naphthalene-1,8-diyl bis(4-*O*-methyl-β-gluco- pyranoside) (**83**) were separated from the mycelia of *C. cicadae* [56].

**Figure 7:**
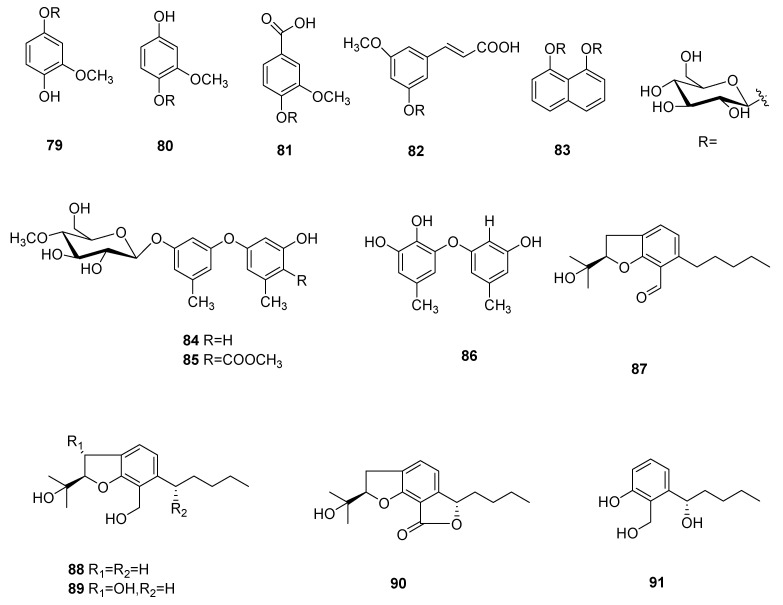
Aromatics and their derivatives (**79**–**91**) from *Cordyceps*.

Cordyols A–C (**84**–**86**) were separated from the *Cordyceps* sp. BCC 1861. Cordyol C (**86**) showed anti-HSV-1 activity, its IC_50_ value was 1.3 μg/mL and it had a cytotoxic effect on BC and NCI-H187 cancer cell lines, whose IC_50_ values were 8.65 μg/mL and 3.72 μg/mL, respectively. Cordyol A (**84**) showed slight antifungal activity and its MIC value was 100 μg/mL [57]. Annullatins A–E (**87**–**91**) were identified from *C. annullata*. Suberoyl bis-hydroxamic acid (SBHA), a histone deacetylase (HDAC) inhibiting agent, was added to the culture medium. Compounds **87** and **88** reduced cAMP levels by 47.9% and 40.8%, respectively, in a CB1-expressing assay. Compounds **87** and **88** decreased cAMP levels by 69.9% and 15.1%, respectively, and compound **89** increased the cAMP standards by 47.5% (CB1 and CB2 were cannabinoid receptors) in CB2-expressing assays [58]. The structures of **79**–**91** are shown in Figure 7.

## 7. Protein

There are also proteins in *C. sinensis*. As far as we know, these proteins have a variety of activities, such as antifungal, anticancer and antiviral activities, etc. These research results show that *C. sinensis* proteins also play an important role in biology. In recent years, studies on *C. militaris* proteins have also been gradually increasing. A new anti-tumor protein was separated from the seed entity of *C. militaris*, named CMIP (**92**). CMIP showed anti-metastasis activity by reducing the amount of tumor nodules in the lung of tumor-bearing mice and extended their lives on a mouse model of 4T1 breast cancer lung metastasis. The results showed that CMIP possessed immune regulatory activity [59].

In addition, a new protein with a molecular mass of 18.0 kDa was separated from *C. militaris* and named CMP (**93**); however, it is a nocuous protein that can cause cell apoptosis via a mitochondrion-dependent mechanism [60]. A glycopeptide (Cs-GP1) (**94**) was purified from the strain *C. sinensis* Cs-HK1, and its molecular weight was 6.0 kDa. Furthermore, Cs-GP1, mostly consisting of glucose and mannose at 3.2:1.0 molar ratio, showed notable antioxidant activities (1183.8 μmol Trolox/g and 611.1 μmol Fe(II)/g) [61]. A protein designated the *Cordyceps militaris* protein (CMP I) (**95**) was the first protein from *C. militaris*; it is a 12 kDa protein in the form of dimers. CMP I exhibited powerful antifungal activities against the growth of the *Fusarium oxysporum*. In the CCK assays, CMP I (95) significantly inhibited the viability of MCF-7 cells, its IC_50_ value was 9.3 μM and the IC_50_ against 5637 cells was 8.1 mΜ. However, CMP I showed almost no inhibitory effect on A-549 cells [62]. Two peptides, named VPRKL(Se)M (Se-P1) (**96**) and RYNA(Se)MNDYT (Se-P2) (**97**), were purified from Se-enriched *C. militaris* and demonstrated neuroprotective effects, and compared to the damage group, Se-P1 and Se-P2 increased PC-12 cell viability by 30 and 33%, respectively. In addition, Se-Ps may moderate cognitive damage in LPS-injured mice (*p* < 0.05). Therefore, Se-Ps has the potential to be an alternative to drugs to prevent and/or treat AD (Alzheimer’s disease) [63].

A fibrinolytic enzyme (**98**) was identified from *C. militaris*, and its enzyme activity was 1682 U/mg; its molecular weight and pI were 32 kDa and 9.3 ± 0.2, respectively; its first-rank pH and temperature were 7.4 and 37 °C, respectively. This fibrinolytic enzyme could hydrolyze fibrin(ogen) rapidly and cleave the α-chains more effectively than β- and γ-chains, and it also could degrade thrombin. Therefore, it could be a potential natural agent for oral fibrinolytic medical treatment or to prevent the formation of blood clots [59]. An antifungal peptide, cordymin (**99**) with a unique N-terminal amino acid sequence was purified from the *C. militaris*; its molecular mass was 10,906 Da. Cordymin inhibited the mycelial growth of *Bipolaris maydis*, *Rhizoctonia solani*, *Mycosphaerella arachidicola* and *Candida albicans* and their IC_50_ values were 50, 80, 10 and 0.75 μM, respectively. Cordymin also had an IC_50_ of 55 μM for inhibiting HIV-1 reverse transcriptase [64]. A novel protease (**100**) was purified and characterised from the edible fungus *C. sinensis*; its molecular weight was approximately 43 kDa; and its first-rate pH and temperature were 9.5 and 30 °C, respectively [65]. A novel fibrinolytic enzyme named CMase (**101**) was purified from *C. militaris* for the first time; its molecular mass was approximated to be 27.3 kDa and its optimal pH and temperature were pH 6.0 and 25 °C, respectively [66]. These reports suggest that *C. militaris* represents a new source of proteins.

## 8. Polysaccharide

Cordyceps polysaccharide is the important bioactive component in *C. sinensis*, which has demonstrated anti-cancer, anti-oxidant, anti-viral, immunomodulation properties and the improvement of liver function [67]. Furthermore, studies have shown that the sulfated exosaccharide of *C. sinensis* can enhance its antioxidant activity [68].

A polysaccharide (SDQCP-1) (**102**) was separated from *C. militaris* that was cultivated on hull-less barley; it mainly consisted of mannose, glucose and galactose at 13.3:1.0:9.7 molar ratio; its average molecular weight was 19.3 kDa. The antioxidant and immunomodulatory activities showed that SDQCP-1 had great antioxidant capacity, its ORACFL value was 24.7 mmol Trolox/g and TEAC value was 202.4 μmol Trolox/g. SDQCP-1 also motivated macrophages to liberate NO, IL-6, TNF-α and IL-10 and mostly facilitated the M1 polarization of macrophages. The findings suggest that SDQCP-1 has potential as a natural antioxidant and immunomodulator in functional foods or drugs [69].

A novel polysaccharide CMP-1 (**103**), with an average molecular weight of 4.3 kDa, was isolated from the fruit body of cultured *C. militaris* with antioxidant, immune stimulatory and anti-tumor activity. CMP-1 showed free radical-scavenging effects, ferrous-ion chelating ability and reducing power in antioxidant assays. Furthermore, CMP-1 considerably encouraged mouse splenocyte proliferation in vitro. It also inhibited the proliferation of HepG2, HeLa, HT-29 and K562 cells and the IC_50_ values were 176.29, 162.59, 137.66 and 364.01 μg/mL, respectively, in cytotoxicity assays [70]. A novel polysaccharide CM3-SII (**104**) was isolated from *C. militaris* with potential hypolipidemic effect; it consisted of mannose, glucose and galactose at a 10.6:1.0:3.7 molar ratio. The interference of CM3-SII considerably increased the protein expression of LDLR and intracellular levels of PCSK9 at the concentration of 100 and 200 μg/mL [71]. A homogeneous exopolysaccharide (EPS-III) (**105**) was obtained from *C.* *militaris* with hypoglycemic activity; its average molecular weight was 1.56 × 10^3^ kDa. In a hypoglycemic experiment of EPS-III in vivo, the inhibition rate of α-glucosidase was considerably enhanced when the concentration of EPS-III was increased, and at a concentration of 3 mg/mL the inhibition rate reached 55.94 ± 1.34%. In addition, studies showed that EPS-III moderated weight loss, decreased plasma glucose concentration, promoted glucose tolerance, secured immune organs and repaired dyslipidemia to moderate diabetes in STZ-induced diabetic mice [72]. An alkaline-extracted polysaccharide (CM3II) (**106**) was purified from *C. militaris* with anti-atherosclerotic effects; it was mainly composed of mannose, glucose and galactose at a 1.4:1.0:1.2 molar ratio. In experimental mice with atherosclerosis induced by a high-fat diet, Oil Red O staining results showed that simvastatin and CM3II interference decreased the atherosclerotic lesion/lumen ratio by 6.1% and 17.8% (*p* < 0.05), respectively. Moreover, CM3II increasingly decreased the TC and TG standards [73]. Two new polysaccharides, SCP II-1 (**107**) and SCP II-2 (**108**), were purified from silkworm *Cordyceps* and demonstrated antioxidant and antitumor activity; the molecular weight of SCP II-1 was 35.2 kDa and SCP II-2 was 23.4 kDa; they consisted of ribose, mannose, glucose and galactose in a molar ratio of 1.0:27.38:8.52:17.99 and 1.0:21.21:1.95:14.28, respectively. In the DPPH radical scavenging activity assays, the DPPH radical removal degrees of SCP II-1 and SCP II-2 were 88.328% and 75.028%, respectively. The DPPH radical removal IC_50_ values were less than 0.5 mg/mL. In the antitumor activity experiment, SCP II-1 had an IC_50_ value of 119.34 ± 1.76 μg/mL against HepG2 cell proliferation [74]. A polysaccharide (CMP-III) (**109**) was isolated from *C. militaris*; its average molecular weight was 4.796 × 10^4^ kDa; it was composed of glucose, mannose and galactose at an 8.09:1.00:0.25 molar ratio. Moreover, the studies of immunomodulatory functions showed that CMP-III could enhance macrophage phagocytosis and the release of NO, TNF-α and IL-6 at a concentration of 25–200 μg/mL [75]. An acidic exopolysaccharide (AESP-II) (**110**) was purified from *C. militaris* that demonstrated immunological activity. AESP-II consisted of mannose, glucuronic acid, rhamnose, galactose acid, N-acetyl-galactosamine, glucose, galactose and arabinose at a 1.07:5.38:1:3.14:2.23:15:6.09:4.04 molar ratio, and its molecular weight was 61.52 kDa. In addition, AESP-II considerably increased the proliferation of B lymphocytes in a dose-dependent manner and significantly increased the proliferation of T lymphocytes at a low dose (25 mg/kg body weight) in an animal experiment [76]. A poly-N-acetylhexosamine (polyhexNAc) (**111**) with an average molecular weight of about 6 kDa was isolated from *C. sinensis* Cs-HK1; its molecular structure is a [-4-β-D-ManNAc-(1 → 3)-β-D-GalNAc-(1 →] disaccharide repeating unit in the chief chain. It exhibited remarkable antioxidant activities (330 μmol Trolox/g and 45.7 μmol Fe(II)/g) and showed meaningful cytoprotective activity at a concentration of 10–200 mg/mL [77]. A novel polysaccharide (CMPA90-1) (**112**) with antioxidant and anti-tumor activity was isolated from *C. militaris*. CMPA90-1 consisted of arabinose, mannose and galactose at a 1.00:2.89:2.03 molar ratio; it exhibited inhibitory activity against A549 cells and its IC_50_ value was 39.08 μg/mL in the cytotoxicity assay [78].

Two novel polysaccharides, PSCK2-2 (**113**) and PSCK2-3 (**114**), which demonstrated good antioxidant properties and powerful protective effects against DNA damage, were isolated from *C. kyushuensis*. PSCK2-2 was composed of Fru-Man-Rha GalN and Ara at a 1.0:1.19:0.11:0.11:0.34 molar ratio, and PSCK2-3 was composed of Fru-Man-Rha Glu and Ara at a 1:1.29:0.14:0.07:0.32 molar ratio. The hydroxyl radical scavenging activities showed that the inhibition rate was 98.33 ± 3.29% (*p* < 0.05) and 55.83 ± 2.41% (*p* < 0.05), respectively, when the concentration of PSCK2-2 and PSCK2-3 was 6 mg/mL; their IC_50_ values were 1.5 mg/mL and 4.8 mg/mL, respectively [79]. *Cordyceps sinensis* polysaccharide 1 (**115**), with a molecular weight of 1.17 × 10^5^ Da, was purified from *C. sinensis*. It consisted of (1 → 6)-linked α-D-Glc and α-D-Gal, with minor β-(1 → 4)-D-Xyl and β-(1 → 4)-D-Man residues presumably seated in the side chains with a trace quantity of α-(1 → 3)-L-Rha residue. In the assays of the restrained proliferation of sarcoma 180 cells, polysaccharide 1 showed significant activity and induced apoptosis in a dosage-dependent manner [80]. A novel polysaccharide CME-1 (**116**) was identified from *C. sinensis*, it was composed of mannose and galactose at a 4:6 molar ratio and its molecular weight was 27.6 kDa. For inhibiting human platelet aggregation, CME-1 with a concentration in the range of 2.3–7.6 μM was highly effective when fueled by collagen, thrombin and arachidonic acid except for U46619 [81].

Two polysaccharides, WIPS (**117**) and AIPS (**118**), were separated from *C. sinensis* Cs-HK1. The molecular weight of WIPS and AIPS were 1.18 × 10^3^ kDa and 1.15 × 10^3^ kDa, and they were elucidated as α-D-glucans with a backbone of (1 → 4)-linked α-D-Glcp (>60%). AIPS inhibited tumor growth by about 28%, and WIPS inhibited about 12% of melanoma tumor growth in mice. In addition, AIPS and WIPS augmented the impact on the T-cell proliferation and viability in the lymphocyte proliferative assay [82]. A novel acidic polysaccharide AEPS-1 (**119**), with immunomodulatory properties, fractionated from the exopolysaccharide produced by *C. sinensis* Cs-HK1, has an α-D-(1 → 3)-Glcp backbone structure. It consists of glucopyranose (Glcp) and pyrano-glucuronic acid (GlcUp) at an 8:1 molar ratio plus a trace amount of mannose; its average molecular weight was about 36 kDa. When the concentration of AEPS-1 was increased from 25 to 250 μg/mL, the cytokine IL-10 also increased but the other three cytokines, TNF-a, IL-1b and IL-6 in the culture medium of macrophage Raw264.7 cells, were decreased [83].

A polysaccharide MCMP (**120**) was purified from *C. militaris* and demonstrated anti-tumor activity. The molecular weight of MCMP was 8.1 kDa, and it consisted of mannose, rhamnose, galactose and glucose at a 59.36:1:8.31:39.50 molar ratio. At a concentration of 8 mg/mL, the inhibition rates of MCMP on HepG-2 cells, Hela cells and mesangial cells were 57.11%, 67.11% and 58.74% after 72 h incubation, respectively [84]. A novel polysaccharide, with a molecular weight of about 82 kDa, named cordysinocan (**121**), was obtained from *Cordyceps*. In cultured T-lymphocytes, cordysinocan induced cell proliferation and its EC_50_ was 6 μg/mL; it also induced IL-2 secretion with an EC_50_ of 8.5 μg/mL. Furthermore, in cultured macrophages, cordysinocan induced macrophage phagocytosis and its EC_50_ was 5 μg/mL; it also increased phagocytosis and the enzymatic efficacy of acid phosphatase [85]. A polysaccharide, namely CBP-1 (**122**), was separated from *C. militaris*. CBP-1 had a backbone of (1 → 4)-α-D-mannose residues, which sometimes branched at O-3. The branches consisted of (1 → 4)-α-D-glucose residues and (1 → 6)-β-D-galactose residues and ended with β-D-galactose residues. CBP-1 demonstrated hydroxyl radical-scavenging activity and its IC_50_ value was 0.638 mg/mL in in vitro antioxidant assays [86].

A novel polysaccharide PS-T80 (**123**) was collected from *Ophiocordyceps sobolifera* and demonstrated antioxidant activities; it was mainly composed of β-D-glucose and α-D-mannose at a 2:1 molar ratio and its average molecular weight was 74 kDa. The structure studies revealed that PS-T80 was a mannoglucan, owing to the repeating unit of [→ 3)-β-D-Glcp-(1 → 3)-α-D-Manp-(1 → 3)-β-D-Glcp-(1 →]. DPPH radical scavenging activity was improved in a dose-dependent manner from a PS-T80 (123) concentration in antioxidant assays, and the IC_50_ value was approximately 0.97 mg/mL [87].

A novel fungal polysaccharide (PS-T100) (**124**) was purified from *Ophiocordyceps sobolifera*; however, its biological activity was not tested. PS-T100 was mainly composed of [→ 3)-β-D-Glcp-(1 → 3)-α-D-Manp-(1 → 3)-β-D-Glcp-(1 → 3)-α-D-Manp-(1 → 3)-β-D-Glcp-(1 →] repeating units; its average molecular weight was 2.29 × 10^2^ kDa [88]. Two novel polysaccharides, CM1 (**125**) and CMS (**126**), were purified from *C. militaris* and demonstrated lipid-lowering activity. CM1 was composed of mannose, glucose and galactose at a 1.4:1.0:1.2 molar ratio, and CMS consisted of glucose. Their molecular weights were 700 kDa and 18.2 kDa, respectively. CM1 mainly consisted of (1 → 4)-β-D-Glcp and (1 → 2)-α-D-Manp residue, and CMS was a homopolysaccharide with → 6)Glcp(1 → linkage. Both CM1 and CMS improved [3H]-cholesterol effusion from macrophages to the medium in a dose-dependent manner (0–100 μg/mL) [89]. A Se-polysaccharide (SeCPS-II) (**127**) was isolated from selenium-enriched *C. gunnii*. Its molecular weight was 4.12 × 10^3^ kDa, and it consisted of α-L-rhamnose, α-D-mannose, α-D-glucose and β-D-galactose at a 4.33:12.62:27.50:18.99 molar ratio. The inhibition rates of SeCPS-II against SKOV-3 cells in the low-, medium- and high-dosage groups were 8.14% (*p* < 0.01), 19.75% (*p* < 0.01) and 36.40% (*p* < 0.01), respectively, in antitumor assays in vivo [90]. A new polysaccharide CMPB90-1 (**128**) with a molecular weight of 5.8 kDa was isolated from cultured *C.* *militaris*; it consisted of Gal, Glc and Man at a 3.04:1.00:1.45 molar ratio. Furthermore, CMPB90-1 considerably increased the proliferation of lymphocytes at a concentration of 31.2–500 µg/mL in splenic lymphocyte proliferation assays and enhanced the killing effect of NK cells on splenocytes in vitro. It also reinforced the phagocytosis influence of macrophages and induced the M1 polarization of the macrophages [91].

A polysaccharide CP2-S (**129**) was purified from *C. militaris*, with a molecular weight of 5.938 × 10^3^ kDa and consisted of glucose. CP2-S significantly stimulated macrophages to take up neutral red, produce NO and increased the excretion of the cytokines IL-1β and IL-6 (50–500 μg/mL) [92]. A protein-polysaccharide HS002-II (**130**) was fractionated from *Hirsutella sinensis* and its average molecular weight was 44 kDa. HS002-II was composed of 57.9% polysaccharide and 42.1% protein and was linked by N-type carbohydrate–protein linkages. It consisted of (1 → 3)-linked α-D-ribofuranosyl units, (1 → 4)-linked α-D-xylopyr-anosyl units and (1 → 4)-linked β-D-glucopyranosyl units. Furthermore, HS002-II induced the expression of pro-inflammatory cytokines TNF-α in the upper clear liquid and IL-1β, NF-κB, TNF-α and iNOS in the transcription level in a concentration-dependent manner (0–2.2 μM) [93]. A new polysaccharide (CM-S) (**131**) was extracted from the fruiting bodies of *C. militaris*. Its molecular weight was 134,631 Da. CM-S consisted of galactose, glucose and xylose at a 3:2:1 molar ratio and its main chain was (1 → 6)-α-d-galactose. In addition, CM-S considerably increased the proliferation of T cells in contrast to the blank control group at 5, 10 and 20 μg/mL [94]. The information of polysaccharides from *Cordyceps* fungi was shown in Table 1.

The fungi of *Cordyceps taii* [95], *Cordyceps gracilioides* [96] and *Cordyceps indigotica* [97,98] taxonomically belonged to *Cordyceps* previously; however, they have now been classified into other genera. Therefore, its metabolites and its metabolites’ biological activities are not described here.

## 9. Conclusions

*Cordyceps sinensis*, a famous and precious traditional Chinese medicine, has various activities, including antitussive, asthma-relieving, immune regulation, antibacterial and antitumor, and has a medicinal history of more than 200 years [1]. Cordycepin, isolated and purified from *Cordyceps*, has also become a focus of research because of its various biological activities. The present review summarizes compounds obtained from *Cordyceps* with new structures or new activities reported from 2007 to 2022. These compounds include nucleosides, non-ribosomal peptides and alkaloids, polyketides and polysaccharides, among others, which show antitumor, antioxidant, antibacterial, hypoglycemic and immune regulation enhancement activities. Furthermore, researchers have also obtained metabolites from *Cordyceps* fungi in different manners. For example, four novel isoflavone methy-lglycosides, daidzein 7-O-β-D-glucoside 4″-O-methylate (**49**), glycitein 7-O-β-D-glucoside 4″-O-methylate (**50**), genistein 7-O-β-D-glucoside 4″-O-methylate (**51**), and genistein 4′-O-β-D-glucoside 4″-O-methylate (**52**) were obtained from *C. militaris* grown on germinated soybeans [46]; two new polyketides cordypyrones A (**68**) and B (**69**) were obtained by the heterologous expression of the gene cluster in *Aspergillus nidulans* [53]; and an acidic exopolysaccharide AESP-II (**110**) was isolated on the basis of the immune activity of the fermentation broth of *C. militaris* [76]. In addition, the paper briefly concludes new activities or new mechanisms of cordycepin that have been reported in recent years. By summarizing the compounds with the new structures and new activities of the known metabolites obtained from the genus *Cordyceps* in the past 15 years, this paper provides a theoretical basis for research on the active compounds of the genus *Cordyceps*.

## 10. Prospects

*Cordyceps* fungi have always attracted scientific attention due to their various biological activities; however, effectively isolating active monomer compounds from them has been challenging. Under laboratory conditions, fungi produce far fewer compounds than they do under natural conditions [99], and through the analysis of fungal genome data and bioinformatics, fungi have a number of biosynthetic gene clusters of active natural products; however, more than 90% are silent [100]. Cordyceps A (**68**) and B (**69**) are novel compounds obtained by heterologously expressing the gene cluster of *C. militaris* in *Aspergillus nidulans*. Therefore, by the method of heterologous expression, the biosynthetic gene cluster of *Cordyceps* fungi can be activated and expressed in filamentous fungi, which can also be used as a potential method for obtaining active compounds in *Cordyceps* fungi. In addition, in recent years some important molecular technologies have been developed in the deep mining of fungal natural products, such as obtaining specific or non-specific target products through molecular genetic manipulation and directly reconstituting the biosynthesis pathway of target compounds in engineered strains to obtain target compounds. The progress of these methods provides technical support for the research of natural products of *Cordyceps* fungi [101]. The research on the metabolites and their activities of *Cordyceps* fungi has been ongoing, and the most in-depth research on the activity and action mechanism has been carried out on cordycepin. In particular, important progress has been made in research on the mechanism of the anti-tumor, immunosuppression and neuroprotective effects of cordycepin [21,22,23,28]. Cordycepin has been clinically studied in multiple clinical settings worldwide as a potential anti-leukemia/anti-cancer chemotherapeutic agent and has passed clinical phase 1 and 2 (clinical trials NCT00003005 (https://clinicaltrials.gov/ct2/show/NCT00003005, accessed on 3 September 2004) and NCT00709215 (https://clinicaltrials.gov/ct2/show/NCT00709215, accessed on 3 July 2008)). Therefore, there is still great research potential and mining value for other active compounds of *Cordyceps* fungi.

## Figures and Tables

**Table 1:** Polysaccharides originated from *Cordyceps* fungi.

Name	Organism Species	Extraction, Medium	M. W.	Bioactivities	References
SDQCP-1	*C. militaris*	Hot water	19.3 kDa	Antioxidant and immunomodulatory	[69]
CMP-1	*C. militaris*	Ultrasound, hot water	4.3 kDa	Antioxidant, immune-stimulatory and anti-tumor	[70]
CM3-SII	*C. militaris*	Alkaline	-	Potential hypolipidemic	[71]
EPS-III	*C. militaris*	Ethanol, Sevag method	1.56 × 10^3^ kDa	Hypoglycemic	[72]
CM3 II	*C. militaris*	Alkaline	-	Anti-atherosclerotic	[73]
SCP II-1	Silkworm *Cordyceps* sp.	Hot water	35.2 kDa	Antioxidant and antitumor	[74]
SCP II-2	Silkworm *Cordyceps* sp.	Hot water	23.4 kDa	Antioxidant	[74]
CMP-III	*C. militaris*	Ultrasound, hot water	4.796× 10^4^ kDa	Immunomodulatory	[75]
AESP-II	*C. militaris*	Ethanol, Sevag method	61.52 kDa	Immunomodulatory	[76]
PolyhexNAc	*C. sinensis*	Ethanol	6 kDa	Antioxidant and cytoprotective	[77]
CMPA90-1	*C. militaris*	Simulated gastric juice, ethanol	-	Antioxidant and anti-tumor	[78]
PSCK2-2	*C. kyushuensis*	Methanol, hot water, ethanol	-	Antioxidant activity and protective effects against DNA damage	[79]
PSCK2-3	*C. kyushuensis*	Methanol, hot water, ethanol	-	Antioxidant and protective effects against DNA damage	[79]
*Cordyceps sinensis* polysaccharide 1	*C. sinensis*	Hot water, ethanol, Sevag method	1.17 × 10^2^ kDa	Antitumor	[80]
CME-1	*C. sinensis*	Double-distilled H_2_O	27.6 kDa	Inhibiting human platelet aggregation	[81]
WIPS	*C. sinensis*	Hot water, alkaline	1.18 × 10^3^ kDa	Antitumor and immunomodulatory	[82]
AIPS	*C. sinensis*	Hot water, alkaline	1.15 × 10^3^ kDa	Antitumor and immunomodulatory	[82]
AEPS-1	*C. sinensis*	Ethanol, Sevag method	36 kDa	Immunomodulatory	[83]
MCMP	*C. militaris*	Hot water, ethanol, Sevag method	8.1 kDa	Antitumor	[84]
Cordysinocan	*Cordyceps* sp.	Ethanol	82 kDa	Immunomodulatory	[85]
CBP-1	*C. militaris*	Ethanol, hot water, alkaline	-	Antioxidant	[86]
PS-T80	*O. sobolifera*	Ethanol, hot water	74 kDa	Antioxidant	[87]
PS-T100	*O. sobolifera*	Hot water, ethanol	2.29 × 10^2^ kDa	-	[88]
CM1	*C. militaris*	Ethanol, hot water	700 kDa	Lipid-lowering	[89]
CMS	*C. militaris*	Ethanol, hot water	18.2 kDa	Lipid-lowering	[89]
SeCPS-II	*C. gunnii*	Hot water, ethanol, Sevag method	4.12 × 10^3^ kDa	Antitumor	[90]
CMPB90-1	*C. militaris*	Water, ethanol, alkaline	5.8 kDa	Immunomodulatory	[91]
CP2-S	*C. militaris*	Hot water, ethanol	5.938 × 10^3^ kDa	Immunomodulatory	[92]
HS002-II	*Hirsutella sinensis*	Papain enzymolysis, Sevag method, ethanol	44 kDa	Immunostimulatory	[93]
CM-S	*C. militaris*	Hot water, ethanol	134631 Da	Immune	[94]

## Data Availability

Not applicable.
